# Evaluating the impact of *Aedes japonicus* invasion on the mosquito community in the Greater Golden Horseshoe region (Ontario, Canada)

**DOI:** 10.1371/journal.pone.0208911

**Published:** 2018-12-13

**Authors:** Camille Dussault, Mark P. Nelder, Curtis Russell, Steven Johnson, Linda Vrbova

**Affiliations:** 1 Centre for Food-borne, Environmental and Zoonotic Infectious Diseases, Public Health Agency of Canada, Toronto, Ontario, Canada; 2 Department of Epidemiology, Biostatistics and Occupational Health, Faculty of Medicine, McGill University, Montreal, Quebec, Canada; 3 Enteric, Zoonotic and Vector-borne Diseases, Communicable Diseases, Emergency Preparedness and Response, Public Health Ontario; Toronto, Ontario, Canada; 4 Analytic Services, Informatics; Knowledge Services, Public Health Ontario, Toronto, Ontario, Canada; Swedish University of Agricultural Sciences, SWEDEN

## Abstract

**Background:**

*Aedes japonicus* was first documented in Ontario, Canada, in 2001. The objective of this study was to determine the effect of *Ae*. *japonicus* establishment on the abundance of other mosquitoes in the Greater Golden Horseshoe (GGH) region of Ontario.

**Methods:**

Adult mosquito data from the Ontario West Nile virus surveillance program were used. Descriptive analyses, linear trends and distribution maps of average trap count per month for six mosquito species of interest were produced. Multivariable negative binomial regression models were constructed to 1) test whether the invasion of *Ae*. *japonicus* affected the abundance of other mosquitoes by comparing the time period before *Ae*. *japonicus* was identified in an area (pre-detection), to after it was first identified (detection), and subsequently (establishment), and 2) identify the variables that explain the abundance of the various mosquito species.

**Results:**

The monthly seasonal average (May–October) of *Ae*. *japonicus* per trap night increased from 2002 to 2016, peaking in September, when the average of most other mosquitoes decrease. There were increased numbers of *Ae*. *triseriatus*/*hendersoni* (Odds Ratio (OR): 1.40, 95% Confidence Interval (CI): 1.02–1.94) and decreased numbers of *Coquillettidia perturbans* (OR: 0.43, 95% CI: 0.26–0.73) in the detection period, compared to the pre-detection period. Additionally, there was a decrease in *Cx*. *pipiens*/*restuans* (OR: 0.87, 95% CI: 0.76–0.99) and *Cq*. *perturbans* (OR: 0.68, 95% CI: 0.49–0.94) in the establishment period, compared to the pre-detection period. None of the most parsimonious explanatory models included the period of the establishment of *Ae*. *japonicus*.

**Conclusions:**

There is no evidence that the introduction of *Ae*. *japonicus* significantly reduced populations of *Ae*. *triseriatus*/*hendersoni*, *Cx*. *pipiens*/*restuans* or *An*. *punctipennis* in the GGH. While further research is needed to understand the impact of the *Ae*. *japonicus* invasion on other mosquito species, our work indicates that, on a regional scale, little impact has been noted.

## Background

The Asian bush mosquito *Aedes japonicus*, native to South Korea and Japan, was first reported in North America from Connecticut, New Jersey and New York in 1998 [1, 2]. Since then, this invasive mosquito has spread rapidly throughout eastern North America and Hawaii, Oregon and Washington (US) [3–5]. The first detection of *Ae*. *japonicus* in Canada occurred in 2001 in southern Quebec and Ontario, followed by New Brunswick (2005), Nova Scotia (2008), Newfoundland (2013) and British Colombia (2014) [6–8].

*Ae*. *japonicus* has been associated with transmission of Japanese encephalitis virus (JEV) in Asia [9]; however, its involvement in transmission of other arboviruses to humans in North America is not well understood. *Ae*. *japonicus* has been demonstrated as a competent vector of eastern equine encephalitis virus (EEEV), JEV, LaCrosse encephalitis virus (LACV), Rift Valley fever virus (RVFV), St. Louis encephalitis virus (SLEV), Cache Valley virus (CVV) and West Nile virus (WNV), under laboratory conditions [9–16]; however, only CVV, LACV, WNV and JEV have been detected in field-collected *Ae*. *japonicus* specimens [9–16]. Of note, laboratory studies have shown that *Ae*. *japonicus* is also a competent vector of chikungunya (CHIKV) and dengue (DENV) viruses [17]. While not considered an important vector in North America, how *Ae*. *japonicus* alters the native mosquito community may have impacts on arbovirus transmission in other vector species.

*Ae*. *japonicus* co-occurs with a variety of other mosquito species within immature stage habitats (e.g., tree holes, rock pools, artificial containers), including *Culex pipiens*, *Culex restuans*, *Anopheles punctipennis*, *Aedes atropalpus* and *Aedes triseriatus* [5–6]. Competition with sympatric species in immature habitats can affect the abundance, fitness and vector capacity of emerging adults [18]. Therefore, it is crucial to understand and study mosquito interactions and mosquito communities in order to predict range and potential ecological, economic and health impacts of an invasive species such as *Ae*. *japonicus* in Ontario.

The success of *Ae*. *japonicus* as an invasive species is largely attributed to the wide range of immature habitats it can colonize, its cold tolerance in all stages, its relatively high genetic diversity via multiple introductions into North America, and long season of adult activity that peaks at opportune times to avoid competition with sympatric species [5]. As with other insect invasions, increased travel, trade and association with humans has contributed to the spread of *Ae*. *japonicus* [19]. *Ae*. *japonicus* possesses desiccation-resistant eggs, an attribute of invasive mosquito species that likely aided its introduction into North America via used automobile tires [20]. In North America, *Ae*. *japonicus* immatures inhabit a wide variety of habitats, including rock pools, catch basins, tires, tree holes, depressions in the soil and other artificial containers made from a variety of materials [5, 21]. Female *Ae*. *japonicus* are primarily mammophilic, feeding on white-tailed deer, Virginia opossums, horses, rodents and humans; however, they will occasionally feed on birds [22–25]. Other invasive mosquitoes, such as *Aedes albopictus*, lend much of their invasive success to their superior competitive edge as larvae over native species [26–27]. The evidence for *Ae*. *japonicus* as a superior competitor is ambiguous; however, there is field evidence of *Ae*. *japonicus* invasion resulting in decreased larval populations of *Ae*. *atropalpus*, *Ae*. *triseriatus* and *Cx*. *restuans* under context-dependent conditions [5, 28–29].

The objective of this study is to determine the effect of *Ae*. *japonicus* establishment on the abundance and distribution of other competing mosquitoes in the Greater Golden Horseshoe region and to describe the mosquito community of southern Ontario. Our null hypothesis is that *Ae*. *japonicus* introduction did not lead to a change in abundance of sympatric mosquito species.

## Methods

### Study location

**Fig 1:**
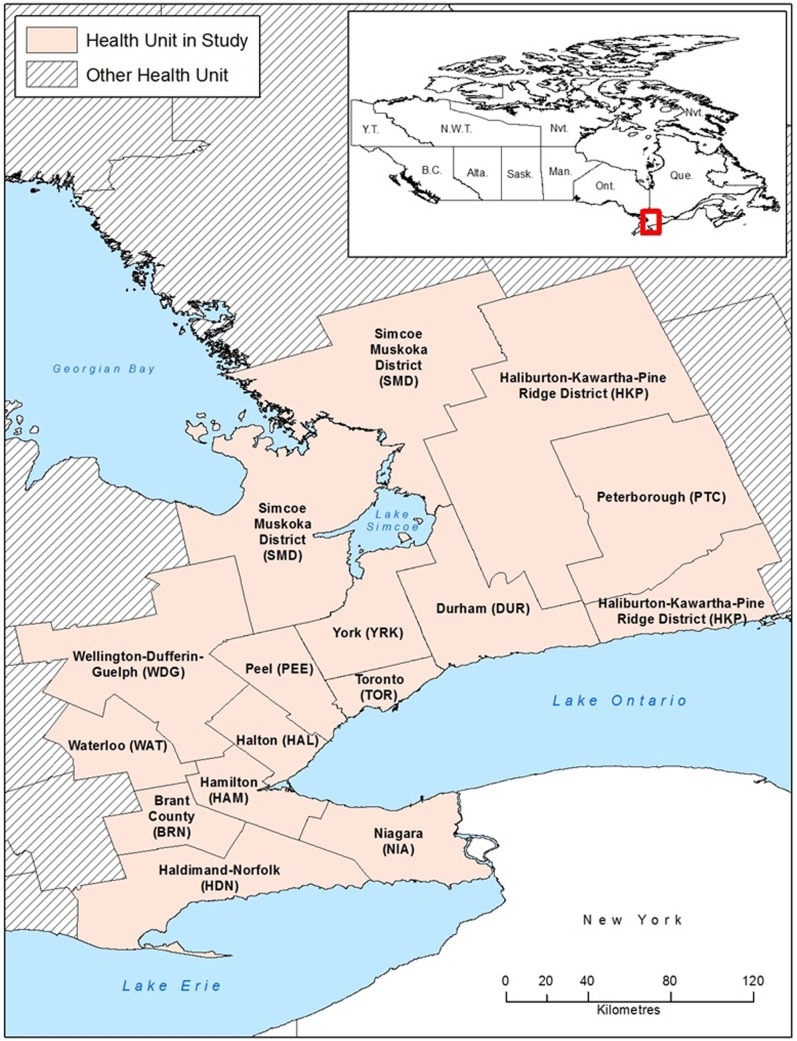
Map of the 14 public health units that form the Greater Golden Horseshoe region in Ontario, Canada.

Ontario, Canada, located in the Great Lakes region of North America is the most populated province in Canada with 13.45 million inhabitants [30]. Ontario is divided into the 36 public health units (PHUs) that administer public health services, including mosquito surveillance. In this study, the Greater Golden Horseshoe (GGH) region (Fig 1) consists of 14 PHUs: Brant County (BRN), Durham Regional (DUR), City of Hamilton (HAM), Halton Regional (HAL), Haldimand-Norfolk (HDN), Haliburton-Kawartha-Pine Ridge District (HKP), Niagara Regional (NIA), Peel Regional (PEE), Peterborough County-City (PTC), Simcoe Muskoka District (SMD), City of Toronto (TOR), Waterloo (WAT), Wellington-Dufferin-Guelph (WDG) and York Regional (YRK). Although the GGH is dominated by a moderate, humid and continental climate with a mix of urban/suburban landscape, there are some rural areas with forests and agricultural land.

### Mosquito collection and identification

Mosquito data that were collected as part of the ongoing WNV surveillance program in Ontario between 2002 and 2016 were used. Adult mosquito collection was done using CO_2_-baited Centers for Disease Control and Prevention (CDC) miniature light-traps with a blacklight, in the 36 PHU areas one night per week, during the WNV season from May to October. PHU staff collected the captured mosquitoes the following morning and sent them to contracted service providers to identify the mosquitoes to species. A subsample of 150 mosquitoes (maximum) collected was randomly identified from each trap, a practice employed by most service providers working in all PHUs. Based on the level of risk, the number of traps a PHU uses can vary from year to year, and even week to week within a season. The number of traps varied from two per week in the low WNV-risk PHUs (e.g., PTC) to as many as 85 per week in the high WNV-risk metropolitan areas (i.e., TOR).

Mosquito collection data are influenced by differences in local surveillance programs, as each PHU implemented these independently. Although the mosquito surveillance program was standardized across Ontario during the study period, the number of mosquitoes identified per trap, for certain years and PHUs, was more than the “maximum” of 150. Since the majority of the service providers stopped identifying mosquitoes after 150 (as was stipulated), including those traps that contained more than 150 mosquitoes would have led to a misrepresentation of the number of mosquitoes identified by certain PHUs and years, representing surveillance artefact rather than higher numbers of mosquitoes. We therefore excluded those traps from further analysis, resulting in 7.1% of data being excluded.

The Ontario mosquito surveillance system targets the primary vectors of WNV, i.e., *Cx*. *pipiens* and *Cx*. *restuans* (host-seeking mosquitoes that primarily feed from dusk to dawn). While not targeting daytime/crepuscular feeding *Aedes* species, such as *Ae*. *japonicus*, the systematic nature of the system should still collect this species uniformly. We assumed that traps in different locations sample the same proportion of the *Ae*. *japonicus* population and that variable rates of capture reflect variable population sizes in the surrounding area. This assumption applied to all other species captured.

### Mosquito species

For this study, sympatric mosquito species refer to the following three species: *Cx*. *pipiens*/ *restuans*, *An*. *punctipennis*, and *Ae*. *triseriatus*/*hendersoni*. We have decided to include these species because 1) they co-occur with *Ae*. *japonicus* in various habitats, and 2) they were the most abundant sympatric species collected in the GGH between 2002–2016. Although *Ae*. *japonicus* invasion led to decreased population of *Ae*. *atropalpus* in several studies in eastern North America, our study did not examine *Ae*. *atropalpus*, as this mosquito is not identified to the species level during routine surveillance [28, 29]. Further work is needed in order to assess the impact of *Ae*. *japonicus* on populations of *Ae*. *atropalpus*.

We have also decided to characterize the impact of *Ae*. *japonicus* introduction on the abundance of the following two allopatric mosquito species: *Coquillettidia perturbans and Aedes vexans*. We chose those two species because 1) we wanted to have control groups, and 2) they were the most abundant allopatric species collected in the GGH between 2002–2016.

### Climate data

Climate data for each PHU were obtained from Natural Resources Canada, produced using spatial climate models generated by thin plate smoothing spline algorithms (ANUSPLIN) [31, 32]. The data included annual historical climate point estimates (2002–2015) for minimum and maximum temperature, precipitation and climate moisture index (CMI) for each of the PHUs of the GGH. We calculated the monthly mean temperature from the monthly average of daily minimum and maximum temperature (°C). Precipitation was the monthly total of the daily precipitation in millimetres. The CMI represents the moisture balance in centimeters, where a positive value denotes an excess of rainfall while a negative value indicates an absence of precipitation.

### Statistical analyses

We used trap count per month as a direct measure of relative female adult mosquito abundance and outcome variable in the analyses. We defined trap count as the number of mosquitoes identified per trap night and averaged for all traps within PHUs by month. We used only traps containing 150 mosquitoes identified or less in the analyses.

To characterize the association between the invasion of *Ae*. *japonicus* and the abundance of other mosquitoes, a time period variable was created for each PHU; time was split into three categories: pre-detection, detection and establishment period. The pre-detection period refers to the time before the first detection of *Ae*. *japonicus*, the detection period refers to the time between the first positive trap to three additional positive traps for *Ae*. *japonicus*, and the establishment period denotes the time after at least four different traps containing *Ae*. *japonicus* were identified. The periods are unique to each PHU, since the various detection dates differ among them. For example, *Ae*. *japonicus* was first detected in Toronto (TOR) in September of 2003, hence the pre-detection period for Toronto is 2002–August 2003, and the detection period starts in September 2003 (S1 Table).

We obtained GGH peer groups data for each of the PHUs of the GGH from the Ontario Ministry of Health and Long-term Care, based on Statistics Canada’s health region peer groups, which uses 24 variables to classify the PHUs by socio-economic characteristics [33]. The urban/rural mix PHUs are characterized by urban-rural mix: BRN, HAM, HDN, HKP, NIA and PTC. We define the urban centre area as PHUs with moderately high population density: DUR, HAL, SMD, WAT and WDG. Metro centre (TOR) and mainly urban PHUs (PEE, YRK) were combined together because they only include one or two PHUs and have a high population density.

Linear regression was used to assess mosquito trends over time for each mosquito species as a function of the year. To test our central hypothesis, univariable (results not shown) and multivariable analyses comparing the overall change in the number of *Cx*. *pipiens*/*restuans*, *Ae*. *triseriatus*/*hendersoni*, *An*. *punctipennis*, *Ae*. *vexans* and *Cq*. *perturbans* per trap night were performed. The distribution of mosquito counts for *Cx*. *pipiens*/*restuans*, *Ae*.*triseriatus*/*hendersoni*, *An*. *punctipennis*, *Ae*. *vexans*, and *Cq*. *perturbans* is highly skewed to the right, due to the high frequency of zero counts. This over-dispersion violates the Poisson model’s assumption that the mean equals the variance, making the Poisson model inappropriate for this data. Therefore, we constructed statistical analyses using a negative binomial model to account for this dispersion. We tested the interaction of variables that changed direction between the univariate and multivariate models. Since none of the interaction terms were statistically significant and the main estimates were similar in the multivariate models with and without the interaction terms, we concluded that there was no evidence of interaction between these variables.

Mosquito abundance for each species was regressed against temperature, precipitation, *Ae*. *japonicus* time period, GGH peer groups and average trap count per month of other mosquitoes independently, followed by a multivariable model that included all of these variables. Since the climate data were only available for 2002–2015, we excluded mosquitoes collected in 2016 from the regression analyses.

We used the Bayesian Information Criterion (BIC) using a forward-backward search to select the best explanatory model for each mosquito species of interest. All analyses were conducted in R v3.2.4.

### Mapping

We aggregated mosquito traps (point locations) into PHUs for mapping and chose map classes that were determined using equal interval classification methods for each set of species maps. We created all maps using ESRI ArcGIS v10.3 software. The data used to map the distribution of the six adult mosquitoes of interest was the seasonal monthly average of mosquitoes per trap night over three 5-year intervals, from 2002 through 2016 by PHUs of the GGH. The 5-year intervals are rough estimates of the pre-detection/detection (2002–2006), establishment (2007–2011), and late establishment (2012–2016) of *Ae*. *japonicus*. Given that there was not a long period of surveillance data available for the pre-detection period in most PHUs, the pre-establishment and establishment time periods were grouped; moreover, a new category of “late establishment” was chosen for the last 5 years of data for illustrative purposes only, as all PHUs in the study area had established populations of *Ae*. *japonicus* as of 2005 (and the time period for “late establishment” is 2012–2016). It should be noted that all of these 5-year time intervals were chosen for illustrative purposes only, given they do not (and cannot) correlate to the statistical analyses: as stated above, each health unit entered into the various establishment phases at a different time. The map intervals are an approximation of when most health units were in the specified interval (e.g. “most” health units in “establishment” interval were in classified as such in the 5-year interval for the statistical analyses).

## Results

**Fig 2:**
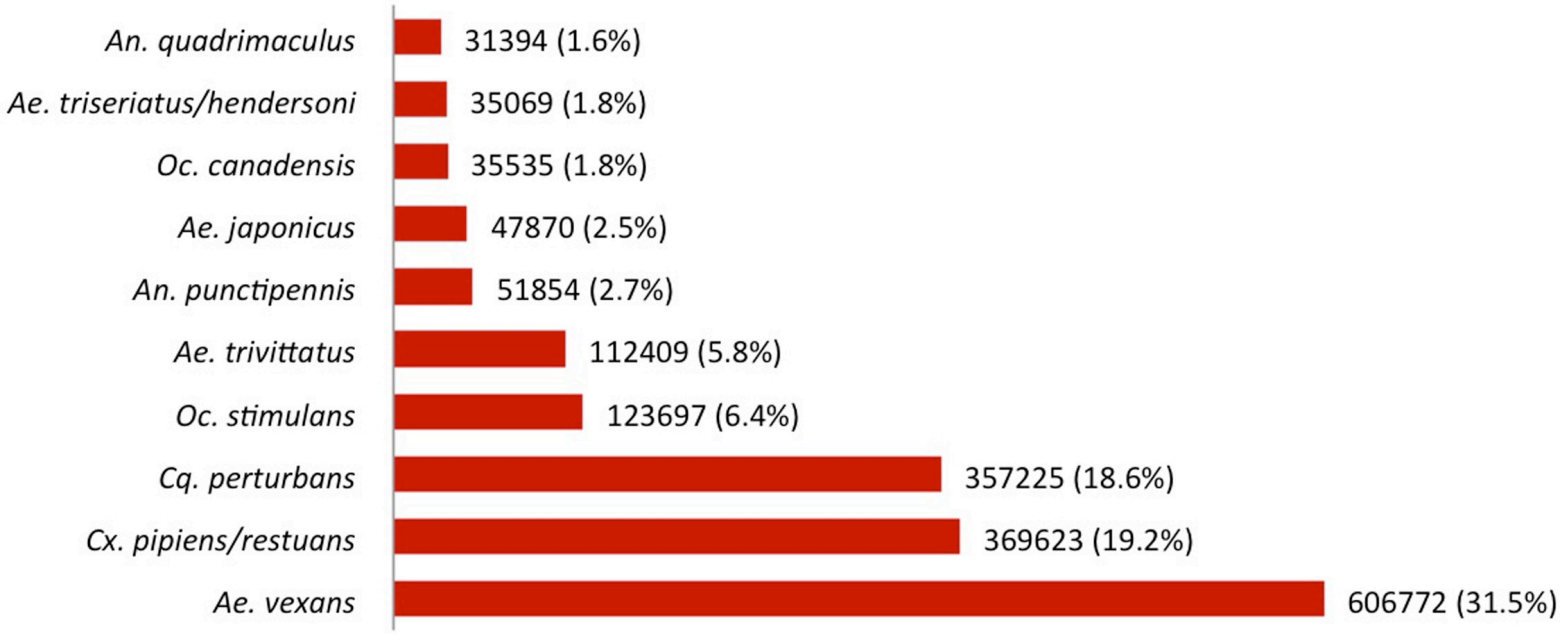
The ten most abundant mosquito species collected in the Greater Golden Horseshoe region, Ontario, Canada (2002–2016).

In the GGH, *Ae*. *japonicus* was detected in two PHUs in 2002 (HAM, NIA), four new PHUs in 2003 (BRN, HAL, PEE, TOR), seven new PHUs in 2004 (DUR, HDN, HKP, PTC, WAT, WDG, YRK) and one new PHU in 2005 (SMD) (S1 Table). Out of the 1,924,395 mosquitoes identified in the GGH between 2002–2016, 47,870 specimens of *Ae*. *japonicus* were collected from 215 (13.3%) trap locations during the study period. The predominant mosquito collected through Ontario’s mosquito surveillance program in the GGH was *Ae*. *vexans*, which represents approximately one third (31.5%) of all mosquitoes identified (Fig 2). *Cx*. *pipiens*/*restuans* (19.2%) was the second most abundant mosquito species, followed by *Cq*. *perturbans* (18.6%), *Ochlerotatus stimulans* (6.4%), *Ochlerotatus trivittatus* (5.8%), *An*. *punctipennis* (2.7%), *Ae*. *japonicus* (2.5%), *Ochlerotatus canadensis* (1.8%), *Ae*. *triseriatus*/*hendersoni* (1.8%) and *Anopheles quadrimaculatus* (1.6%) (Fig 2).

**Fig 3:**
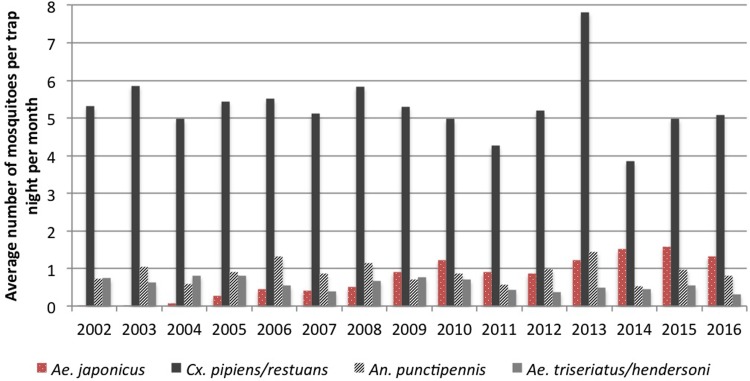
Seasonal monthly average number of mosquitoes per trap night in the Greater Golden Horseshoe region, Ontario, Canada (2002–2016). Seasonal monthly average number of mosquitoes per trap night refers to the monthly average of mosquitoes over the WNV season (May–Nov) each year.

The average number of mosquitoes collected per trap night differed widely between mosquito species, PHUs and years (Table 1). The monthly average of *Ae*. *japonicus* per trap night has been increasing almost every year between 2002–2016, with a range of 0.001 (2002) to 1.69 (2015) specimens in the GGH (Fig 3), and this trend was statistically significant (slope: 0.11/year, p<0.001). Conversely, the monthly average number of *Ae*. *triseriatus*/*hendersoni*, which appears to have decreased over the years, has a statistically significant trend (slope: -0.03/year, p<0.001). The monthly mosquito average for *Cx*. *pipiens*/*restuans* (slope: -0.02/year, p = 0.56) and *An*. *punctipennis* (slope: 0.001/year, p = 0.91) appears relatively stable during the 15 years.

**Table 1:** Summary statistics for the mosquito dataset that contain ≤150 mosquitoes identified, Ontario, Canada (2002–2016).

Public health Unit	Average number of female mosquitoes identified per trap night						No. Trap locations	% Trap excluded
	*Ae*. *japonicus*	*Cx*. *pipiens*/ *restuans*	*Ae*. *triseriatus*/ *hendersoni*	*An*. *punctipennis*	*Ae*. *vexans*	*Cq*. *perturbans*		
**BRN**	2.48	5.16	1.19	2.05	10.22	4.51	28	5.0
**DUR**	0.30	7.69	0.36	0.61	12.79	15.06	53	6.3
**HAL**	1.08	9.07	1.22	1.69	13.86	5.62	127	2.5
**HAM**	0.66	5.55	0.30	0.59	6.99	1.55	71	6.4
**HDN**	0.42	4.98	0.57	1.25	10.47	2.50	49	7.9
**HKP**	0.38	2.71	0.30	0.72	7.69	13.54	150	13.2
**NIA**	0.82	5.60	0.17	0.64	13.17	1.62	64	2.0
**PEE**	1.53	9.73	1.38	1.36	15.36	6.83	356	12.2
**PTC**	0.57	4.22	0.30	0.73	8.91	27.26	51	17.6
**SMD**	0.37	3.68	0.46	0.53	6.30	14.96	53	3.6
**TOR**	0.95	9.21	0.58	0.64	11.36	2.47	292	5.0
**WAT**	1.27	3.19	0.90	1.57	10.14	6.32	196	11.2
**WDG**	0.17	3.82	0.69	0.98	5.01	6.60	35	12.7
**YRK**	0.32	4.06	0.16	0.48	8.55	6.21	94	4.3

**Fig 4:**
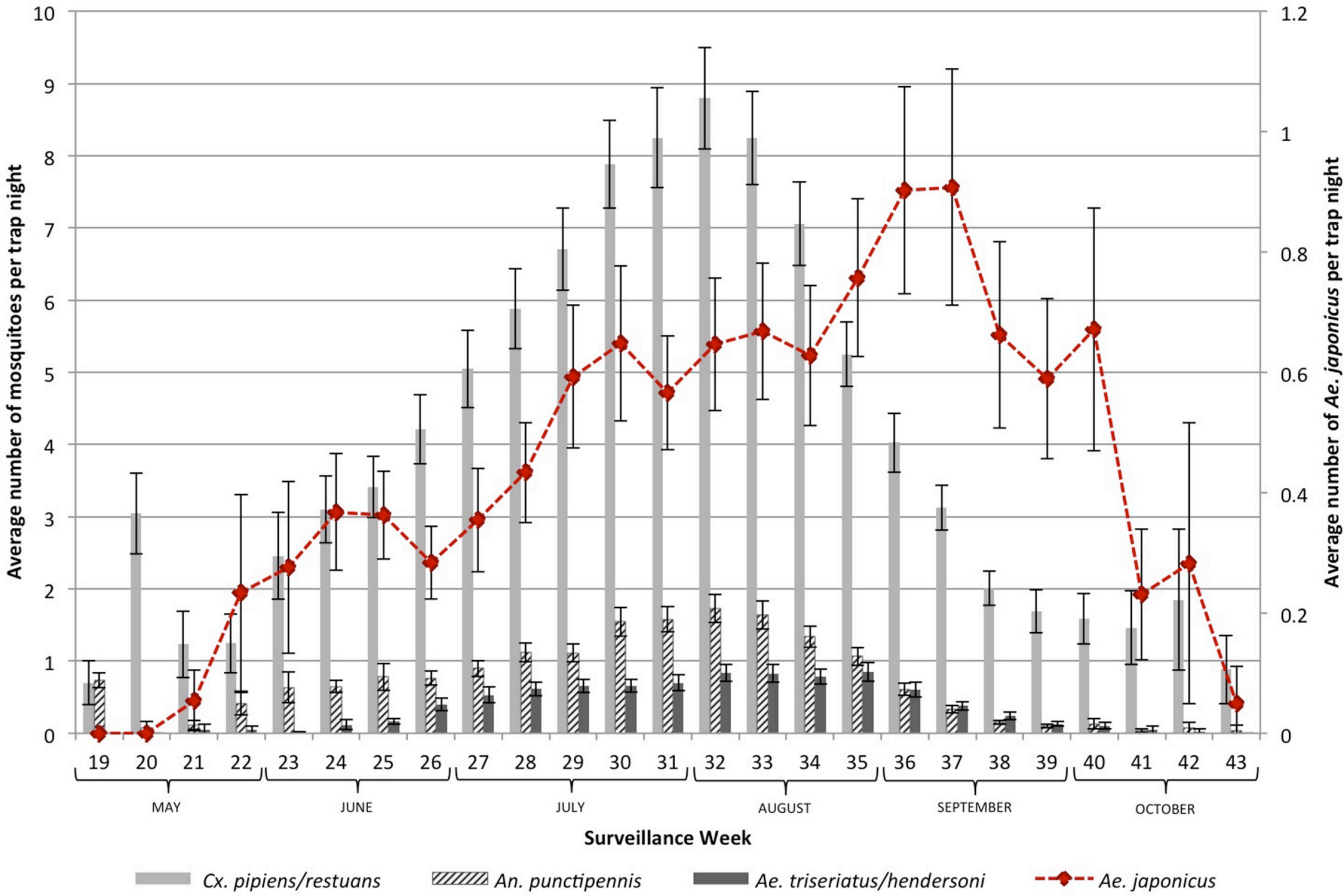
Seasonality of sympatric mosquito species of interest in the Greater Golden Horseshoe, Ontario, Canada (2002–2016). Container species compared to *Ae*. *japonicus*.

**Fig 5:**
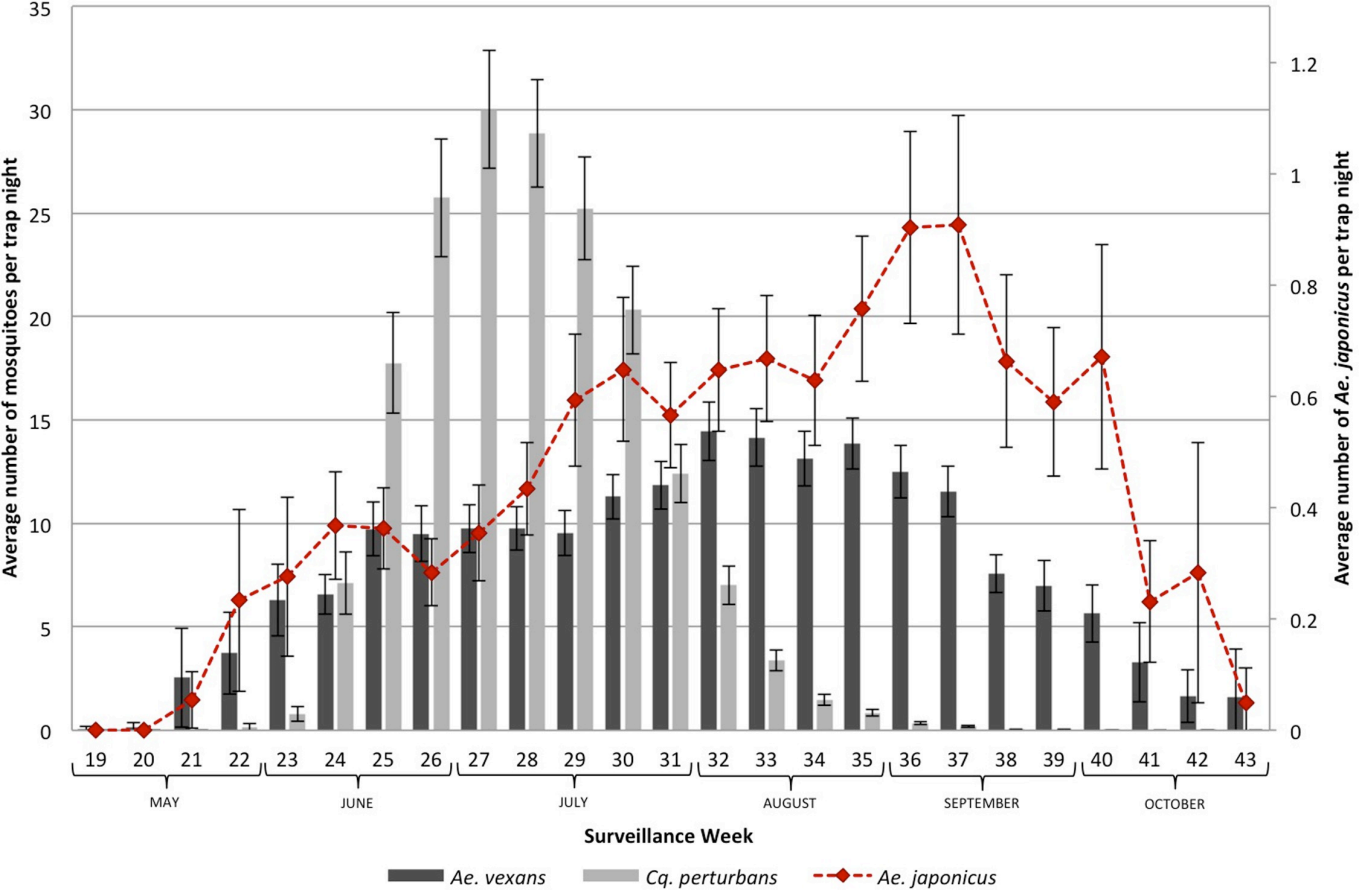
Seasonality of allopatric mosquito species of interest in the Greater Golden Horseshoe, Ontario, Canada (2002–2016). Non-container species compared to *Ae*. *japonicus*.

Figs 4 and 5 show the seasonality of the six mosquito species of interest in the GGH during the study period. Fig 4 shows the three species that share the same ecological niche as *Ae*. *japonicus*, whereas Fig 5 illustrates the two species with different ecological niches. The peak months for *Cx*. *pipiens*/*restuans*, *Ae*. *triseriatus*/*hendersoni* and *An*. *punctipennis* are July and August, while *Ae*. *japonicus* peaks in September, when the average of the other mosquitoes decreases (Fig 4). *Cx*. *pipiens*/*restuans* is also present in early May and is the most abundant species throughout the surveillance season. June and July are the peak months for *Cq*. *perturbans*, while August and September have the highest number of *Ae*. *vexans* and *Ae*. *japonicus* (Fig 5).

**Fig 6:**
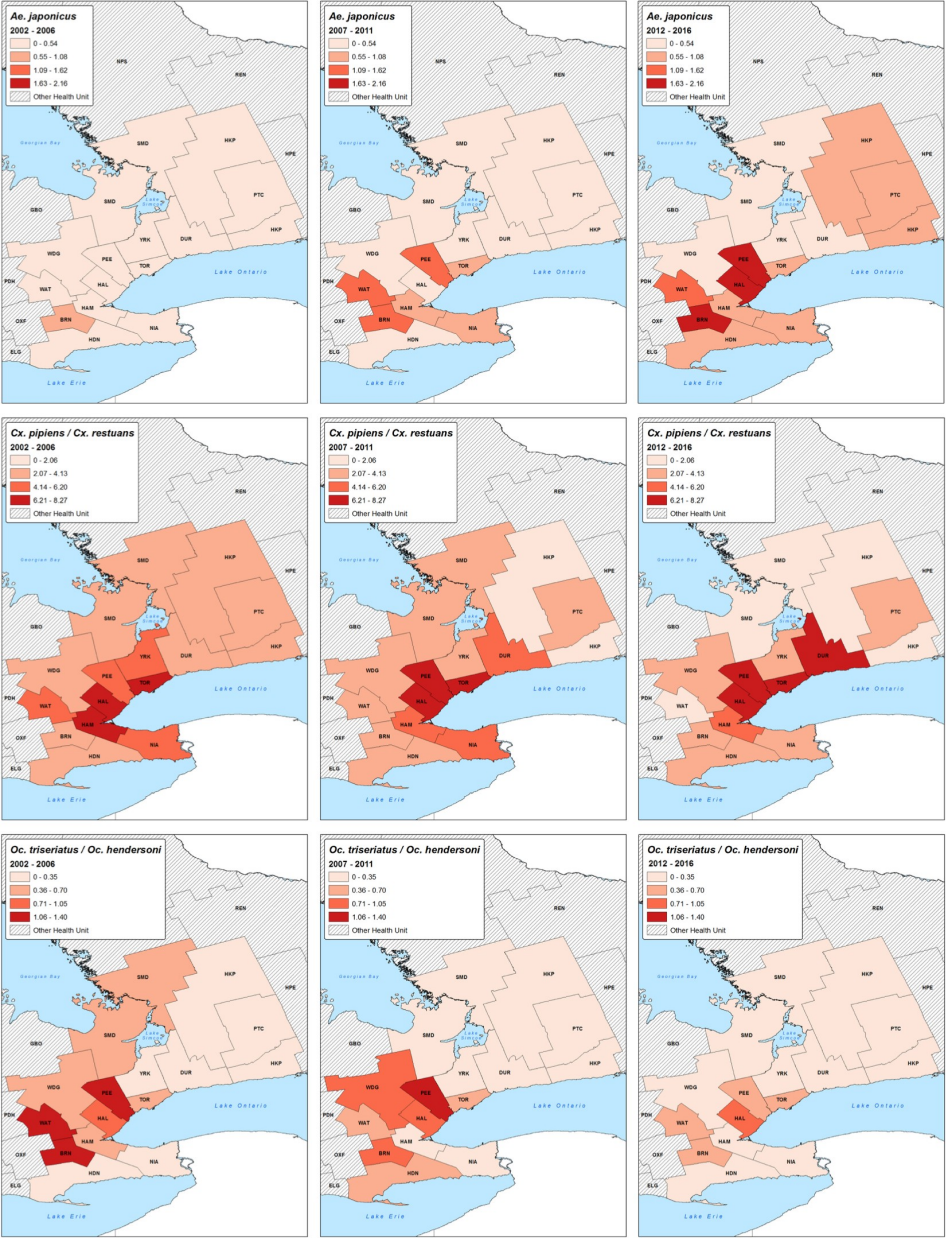
Distribution of mosquitoes in the Greater Golden Horseshoe, Ontario, Canada (2002–2016). The data used to map the distribution of these six adult mosquitoes was the seasonal monthly average of mosquitoes per trap night over three 5-year interval periods, from 2002 through 2016 by PHUs of the GGH. The 5-year intervals are rough estimates of the pre-detection/detection (2002–2006), establishment (2007–2011), and late establishment (2012–2016) of *Ae*. *japonicus* to illustrate its invasion.

Fig 6 displays descriptive maps of the distribution of the six mosquito species by PHUs of the GGH in three 5-year intervals roughly correlating with pre-detection/detection (2002–2006), establishment (2007–2011) and late establishment (2012–2016) of *Ae*. *japonicus*. The number of PHUs with a growing population of *Ae*. *japonicus* increased over the three time periods: in 2002–2006, there was no PHU in the two highest average cut-offs (1.09 to 2.16 mosquitoes per trap), while in 2012–2016, there were three (BRN, HAL, PEE). The distribution of *Ae*. *triseriatus*/*hendersoni* changed over the three time periods, with a reduction of three to zero PHUs in the highest cut-off (1.06 to 1.40 mosquitoes per trap). Overall, the distribution of *Cx*. *pipiens*/*restuans* remained similar between the three periods, with HAM, HAL, PEE, TOR and DUR having a consistently high mosquito average. The distribution of *An*. *punctipennis* decreased in WDG, DUR and BRN, but remained high in WAT, HAL, PEE and HDN. The population of *Ae*. *vexans* appeared to be declining in the majority of the GGH PHUs. On the other hand, the distribution of *Cq*. *perturbans* grew in the northeastern GGH, especially in HKP, PTC and DUR.

Table 2 reports the estimates of the negative binomial regression models for *Cx*. *pipiens*/*restuans*, *Ae*. *triseriatus*/*hendersoni*, *An*. *punctipennis*, *Ae*. *vexans* and *Cq*. *perturbans* with the number of trap nights as the offset parameter. The reference category of the multivariable model is the pre-detection period in metro centre/urban areas. By individual mosquito species, the multivariable model (Table 2) suggests that, when all other variables remain constant and in comparison to the pre-detection period, the detection and establishment periods had on average: 4% more and 13% fewer number of *Cx*. *pipiens*/*restuans* per trap night, 18% fewer and 10% more number of *An*. *punctipennis* per trap night, 40% more and 7% fewer number of *Ae*. *triseriatus*/*hendersoni* per trap night, 18% fewer and 4% more number of *Ae*. *vexans* per trap night, and 57% and 32% fewer number of *Cq*. *perturbans* per trap night. However, only the detection estimates of *Ae*. *triseriatus*/*hendersoni* (odds ratio (OR): 1.40, 95% Confidence Interval (CI): 1.02–1.94) and *Cq*. *perturbans* (OR: 0.43, 95% CI: 0.26–0.73) and the establishment estimate of *Cx*. *pipiens*/*restuans* (OR: 0.87, 95% CI: 0.76–0.99) and *Cq*. *perturbans* (OR: 0.68, 95% CI: 0.49–0.94) were statistically significant.

**Table 2:** Negative binomial regression summary of establishment period as a predictor of mosquito abundance for the Greater Golden Horseshoe region, Ontario, Canada.

Variable	Multivariable Model^b^	
	Coefficient (OR)	95% CI
**Pre-detection**^**a**^	1.00	–
**Detection**		
*Cx*. *pipiens*/*restuans*	1.04	0.86–1.28
*An*. *punctipennis*	0.82	0.64–1.05
*Ae*. *triseriatus*/*hendersoni*	1.40^c^	1.02–1.94
*Ae*. *vexans*	0.82	0.64–1.06
*Cq*. *perturbans*	0.43^c^	0.26–0.73
**Establishment**		
*Cx*. *pipiens/restuans*	0.87^c^	0.76–0.99
*An*. *punctipennis*	1.10	0.94–1.28
*Ae*. *triseriatus*/*hendersoni*	0.93	0.75–1.14
*Ae*. *vexans*	1.04	0.88–1.22
*Cq*. *perturbans*	0.68^c^	0.49–0.94

Dataset containing trap with 150 or less mosquitoes identified was used to populate this table.

^a^The reference category of the multivariable model is Pre-detection.

^b^All values of the estimates above have been adjusted for the following variables: temperature, precipitation, CMI, GGH peer groups and average number of the four other mosquitoes.

^c^ Statistically significant at p<0.05.

The best model for the forward-backward procedure that minimizes the BIC criterion for the GGH dataset differed among the five mosquitoes of interest (Table 3). The best model for *Cx*. *pipiens*/*restuans* was a model with six variables (plus intercept): mean temperature, precipitation, CMI, GGH peer groups and the average number of *An*. *punctipennis* and *Ae*. *vexans*, while the model for *An*. *punctipennis* contained seven variables (plus intercept): mean temperature, precipitation, CMI, GGH peer groups and the average number of *Cx*. *pipiens*/*restuans*, *Ae*. *triseriatus*/*hendersoni* and *Ae*. *vexans*. Although *Ae*. *triseriatus*/*hendersoni* and *Ae*. *vexans* also contained six variables (plus intercept), the variables were different as the first mosquito includes the average of *Cx*. *pipiens*/*restuans* instead of the mean temperature and the later mosquito included all four mosquito averages rather than the precipitation. It is noteworthy to mention that none of these five most parsimonious models included the time period variable of the establishment of *Ae*. *japonicus*.

**Table 3:** Model selection results using BIC forward-backward search for each mosquito species of the Greater Golden Horseshoe region, Ontario, Canada.

Variable	*Cx*. *pipiens*/ *restuans*	*An*. *punctipennis*	*Ae*. *triseriatus*/ *hendersoni*	*Ae*. *vexans*	*Cq*. *perturbans*
**Time period**	X	X	X	X	X
**Temperature**	✓	✓	✓	✓	X
**Precipitation**	✓	✓	X	X	✓
**Climate Moisture Index**	✓	✓	✓	✓	✓
**GGH peer groups**	✓	✓	✓	X	X
***Cx*. *pipiens*/ *restuans***	–	✓	✓	✓	X
***An*. *punctipennis***	✓	–	✓	✓	✓
***Ae*. *triseriatus*/ *hendersoni***	X	✓	–	✓	X
***Ae*. *vexans***	✓	✓	✓	–	✓
***Cq*. *perturbans***	X	X	X	✓	–

Dataset containing trap with 150 or less mosquitoes identified was used to populate this table.

✓ Statistically significant variable (p<0.05) that was included in the most parsimonious explanatory model.

X Non-statistically significant variable (p<0.05) that was not included in the most parsimonious explanatory model.

## Discussion

In this study, we examined the effect of the invasion of *Ae*. *japonicus* on the average of other mosquitoes, taking into account GGH peer groups and climate variables, using the Ontario’s mosquito surveillance data. We found little evidence to suggest that the invasion of *Ae*. *japonicus* has led to a change in the adult mosquito population in the GGH.

While there were statistically significant relationships associated with *Ae*. *japonicus* invasion and the abundance of other mosquito species, these relationships are neither ecologically relevant, nor easily explained. There were marginally significant results for *Cx*. *pipiens*/*restuans* in the establishment phase only, where *Ae*. *japonicus* had a negative impact. In contrast, *Ae*. *triseriatus/hendersoni* was statistically significant for the detection period only, where *Ae*. *japonicus* had a positive effect. If *Ae*. *japonicus* was a superior competitor, we should expect to see a decrease in the number of sympatric mosquito species in both time periods of *Ae*. *japonicus* establishment in the GGH. While there are some statistical significant results from the regression analyses, they are not consistent across sympatric species, nor are they consistent for particular species in both detection and establishment phases. The difficulty in interpreting our results could be remedied in future studies by testing our hypotheses at smaller scales. The surveillance of larvae in these container habitats, compared to adult mosquito surveillance, would be more advantageous for the detection of competition and displacement involving *Ae*. *japonicus*. For example, occupation of containers is heterogeneous, with some containing only native species, others only *Ae*. *japonicus*, and others with mixtures of species; even with competition and displacement occurring, the result may lead to no overall change in the numbers of species being compared.

There is only another one study that has explored the impact of *Ae*. *japonicus* on adult mosquito populations in North America, specifically in New Jersey [34]. Similar to our findings, the invasion of *Ae*. *japonicus* did not have a negative impact on native *Ae*. *triseriatus* adult populations, indicating negligible interspecific competition between the species. Although the multivariable regression shows that the detection period is significantly associated with a 40% increase in the number of *Ae*. *triseriatus*/*hendersoni* compared with the pre-detection period, the time period of *Ae*. *japonicus* establishment was not selected in the most parsimonious model. Indeed, the best predictors for the relative abundance of *Ae*. *triseriatus*/*hendersoni* are temperature, CMI, GGH peer groups and other mosquitoes, suggesting that the time period variable does not drive the population of *Ae*. *triseriatus*/*hendersoni*. It is not surprising that the temperature and moisture index are positively associated with the number of mosquitoes, as those environmental variables play a key role in the aquatic and adult stages of the mosquito life cycle.

In addition, our study has showed that *Ae*. *japonicus* has not affected the abundance of *Ae*. *vexans* but had a significant negative association with *Cq*. *perturbans* in both detection and establishment period. These two species were used in this study as “controls,” since *Ae*. *vexans* and *Cq*. *perturbans* do not occupy the same ecological niche (container habitats) as *Ae*. *japonicus* and thus, as they do not compete for breeding sites, we expected no influence on the mosquito abundance in either time period of both mosquito species. *Ae*. *vexans* is a floodwater mosquito (temporary woodland pools or irrigation fields), while *Cq*. *perturbans* occupies marshes and swamps, which explains why these species do not have the same association with the establishment of *Ae*. *japonicus* [35]. The negative association of *Ae*. *japonicus* and *Cq*. *perturbans* is not the result of competition, but rather the two species are responding differently to environmental and habitat variables examined in this study.

Similar to the seasonality of *Ae*. *japonicus* reported previously by others, the majority of *Ae*. *japonicus* specimens were collected in August and September [6, 21]. However, the number of mosquitoes per trap night for *Cx*. *pipiens*/*restuans*, *Ae*. *triseriatus*/*hendersoni* and *An*. *punctipennis* were higher in July and August decreasing in September, suggesting that *Ae*. *japonicus* segregates itself in adult mosquito populations (in terms of emergence). Indeed, there is no evidence for a significant reduction in population numbers of *Ae*. *triseriatus*/*hendersoni*, *Cx*. *pipiens*/*restuans* or *An*. *punctipennis*. Furthermore, *Ae*. *japonicus* thrives in the absence or declining populations of species with similar ecological niche. It may be that the seasonality of *Ae*. *japonicus* has expanded further in the fall to avoid competition with other host-seeking adult mosquitoes, which can explain in part the increase in mosquito abundance of *Ae*. *japonicus* in GGH. If adult populations of mosquitoes are indeed a proxy for immature competition, then there is no evidence of competitive reduction or exclusion impacts on Ontario’s native mosquito populations, at least at scales studied here.

From its introduction in 2001, *Ae*. *japonicus* spread rapidly across the province, occupying 36 out of 36 PHUs by 2013. Given the poor flight capabilities of this mosquito, and relatively rapid expansion, *Ae*. *japonicus* was likely aided by human transport, either in transport as adults in vehicles or more likely transport of artificial containers containing eggs [4, 20]. The mosquito surveillance data for Ontario indicate, putatively, that there were likely two independent introduction of *Ae*. *japonicus* in Ontario. The first introduction centered in the Niagara Peninsula (HAM, NIA) in 2002 [6]. The second introduction was centered in the eastern portion of the province (OTT, LGL). The timing of arrival of *Ae*. *japonicus* in the Niagara Peninsula (NIA, HAM; 2001–2003) coincides with the first detection of the species in neighboring Erie County, New York (Buffalo) in 2000 [36]. In addition, the timing for PHUs in eastern Ontario (HPE, LGL, OTT; 2003) coincides with detections in Jefferson Co., NY in 2000. Further research is needed to elucidate the exact number of introductions and to identify the source populations for these introductions, i.e., phylogenetic analysis of the COI sequences.

Although the descriptive maps do not allow us to make any conclusion between the distribution of mosquitoes and the establishment of *Ae*. *japonicus*, they allow us to visualize the distribution of each mosquito species by PHU of the GGH over time. The maps suggest that there is a decrease in the abundance and distribution of *Ae*. *triseriatus*/*hendersoni*, as all PHU from the urban centre area, (except HAL), changed to a lower cut-off classification over time. This decrease was confirmed by a statistically significant monthly trend, suggesting that there is a small reduction in the population of *Ae*. *triseriatus*/*hendersoni* over time. The distribution and abundance of *Cx*. *pipiens*/*restuans*, *An*. *punctipennis* and *Ae*. *vexans* in the GGH appeared stable during the study period, not reflecting competition between sympatric species. Once more, the most interesting finding is regarding *Cq*. *perturbans*, where there is an increase in the mosquito average in the northeast region of the GGH. This is surprising because first, we hypothesized no change in the abundance and distribution of allopatric species, and second, the regression analysis showed that the detection and establishment period of *Ae*. *japonicus* was negatively associated with the number of *Cq*. *perturbans*. Many other factors such as climate and urbanization can influence the range and occurrence of mosquitoes. As such, the negative association between *Ae*. *japonicus* and *Cq*. *perturbans* might simply be the results of an indirect association caused by some underlying environmental changes that occurred in the GGH.

There is a need to further investigate mosquito interactions and to evaluate long-term impact of invasive species on sympatric species as well as on allopatric species.

### Limitations

While the mosquito surveillance data used in this study are the most complete and comprehensive for Ontario, there are several limitations associated with the data and the analyses performed. The dataset contained data entry errors for date and geographic location fields. For 0.49% of records, the month and day for the collection date were reversed; fortunately, the dates could be corrected by using the surveillance week. A small proportion (0.83%) of GPS coordinates did not match the PHU. The overall PHU field was considered more accurate than the longitudinal and latitudinal fields, as those fields were more sensitive to entry errors from the service providers and to GPS inaccuracy. Although the temperature and precipitation can differ between trap locations within the same PHU, we chose only one GPS location in each PHU to obtain the climate point estimate due to these GPS errors.

As mentioned previously, we excluded traps that contained over 150 mosquito identified from the analysis due to potential surveillance artefact, resulting in excluding 7.1% of the data. While we decided to exclude these traps from the analysis, it would be interesting for future studies to include them (e.g. by transforming data into a format allowing for simple random sampling from each trap over 150). Since location and the number of traps changed over time and within PHUs, we decided against calculating and testing the proportion of catches for each species in each trap over time to avoid misleading and biased results. Although we used the average number of mosquitoes per trap night in the analyses and used the number of traps as the offset parameter in the regression models, other surveillance artefacts, such as mosquito control effort and resources, as well as other unmeasured differences among PHUs, need to be addressed in further studies.

We aggregated mosquito abundance data and climate data to monthly averages, which can reduce the impact of those variables in the analysis. We excluded 2016 data from the regression analyses because the climate data were not yet available for that year. However, this should not have a huge impact on the outcome since the establishment of *Ae*. *japonicus* in GGH occurred in early 2000’s, and the 14 years of data included should capture any invasion effects. Finally, the CDC light traps used, which target mosquitoes that are active from dusk to dawn, have been reported to be inferior to gravid traps in terms of capturing *Ae*. *japonicus* [6].

## Conclusions

If *Ae*. *japonicus* is a superior competitor, we should have expected to see decreases in sympatric species; however, there was no consistent evidence for a significant reduction in population numbers of *Ae*. *triseriatus*/*hendersoni*, *Cx*. *pipiens*/*restuans* or *An*. *punctipennis*. While further research is needed to better understand the impact of the *Ae*. *japonicus* invasion on other mosquito species, our work indicates that on a regional scale little impact has been noted.

## Supporting information

S1 Table Initial dates of *Aedes japonicus* invasion in Ontario’s 36 public health units. (DOCX)

## Abbreviations

BIC: Bayesian Information Criterion
BRN: Brant County
CDC: Centers for Disease Control and Prevention
CHIKV: chikungunya virus
CMI: climate moisture index
CVV: Cache Valley virus
DENV: dengue virus
DUR: Durham Regional
EEEV: eastern equine encephalitis virus
GGH: Greater Golden Horseshoe region
HAL: Halton Regional
HAM: City of Hamilton
HDN: Haldimand-Norfolk
HKP: Haliburton-Kawartha-Pine Ridge District
JEV: Japanese encephalitis virus
LACV: LaCrosse encephalitis virus
NIA: Niagara Regional
PEE: Peel Regional
PTC: Peterborough County-City
RVFV: Rift Valley fever virus
SLEV: St. Louis encephalitis virus
SMD: Simcoe Muskoka District
TOR: City of Toronto
WAT: Waterloo
WDG: Wellington-Dufferin-Guelph
WNV: West Nile virus
YRK: York Regional
