# Supplementary material for: Evaluating the impact of Aedes japonicus invasion on the mosquito community in the Greater Golden Horseshoe region (Ontario, Canada)
Source: PLoS One. 2018 Dec 13;13(12):e0208911. doi: 10.1371/journal.pone.0208911 (PMC6292619; doi:10.1371/journal.pone.0208911)
Supplement: S1 Table — (DOCX) [file pone.0208911.s001.docx]

**Additional file 1.** Initial dates of *Aedes japonicus* invasion in Ontario’s 36 public health units

| **Public health unit** | **Abbreviation** | **Date first detected** |
| --- | --- | --- |
| Hamilton | HAM | 08/08/2002 |
| Niagara Region | NIA | 08/14/2002 |
| Halton Region | HAL | 07/30/2003 |
| Ottawa | OTT | 08/13/2003 |
| Leeds, Grenville and Lanark District | LGL | 08/14/2003 |
| Brant County | BRN | 08/26/2003 |
| Hastings and Prince Edward County | HPE | 09/03/2003 |
| Peel | PEE | 09/25/2003 |
| Toronto | TOR | 09/25/2003 |
| Eastern Ontario | EOH | 06/17/2004 |
| Middlesex-London | MSL | 06/23/2004 |
| Haldimand-Norfolk | HDN | 07/07/2004 |
| Elgin-St. Thomas | ELG | 07/13/2004 |
| York Region | YRK | 07/20/2004 |
| Region of Waterloo | WAT | 07/21/2004 |
| Haliburton, Kawartha, Pine Ridge District | HKP | 07/28/2004 |
| Oxford County | OXF | 07/29/2004 |
| Perth District | PDH | 08/17/2004 |
| Chatham-Kent | CHK | 08/24/2004 |
| Durham Region | DUR | 08/25/2004 |
| North Bay Parry Sound District | NPS | 08/31/2004 |
| Windsor-Essex County | WEC | 09/15/2004 |
| Peterborough | PTC | 09/22/2004 |
| Wellington-Dufferin-Guelph | WDG | 09/22/2004 |
| Kingston, Frontenac, Lennox & Addington | KFL | 06/14/2005 |
| Renfrew County & District | REN | 06/21/2005 |
| Huron County | HUR | 07/05/2005 |
| Lambton | LAM | 07/13/2005 |
| Simcoe Muskoka District | SMK | 08/18/2005 |
| Grey Bruce | GBO | 09/13/2005 |
| Sudbury & District | SUD | 08/09/2007 |
| Algoma | ALG | 07/28/2009 |
| Thunder Bay District | THB | 08/12/2009 |
| Timiskaming | TSK | 08/18/2009 |
| Northwestern | NWR | 08/03/2011 |
| Porcupine | PQP | 07/23/2013 |
